# Emergent Management of a Tracheoinnominate Fistula in the Community Hospital Setting

**DOI:** 10.7759/cureus.8403

**Published:** 2020-06-02

**Authors:** Abhiram Kondajji, Agnieszka Dombrowska, Matthew Allemang, Thomas Santoscoy

**Affiliations:** 1 General Surgery, Cleveland Clinic South Pointe Hospital, Warrensville Heights, USA; 2 General Surgery/Breast Surgery, Samaritan Medical Center, Watertown, USA; 3 Digestive Disease and Surgical Institute, Cleveland Clinic, Cleveland, USA; 4 Heart, Vascular, Thoracic Institute, Cleveland Clinic, Cleveland, USA

**Keywords:** tracheoinnominate fistula, utley, tracheostomy, herald bleed

## Abstract

Tracheoinnominate fistula is a rare but highly lethal complication of tracheostomy. Early recognition and interventions are key to patient survival.

A 63-year-old woman had undergone tracheostomy for respiratory failure secondary to disseminated histoplasmosis. She presented to the community hospital intensive care unit from a long-term acute care facility for presumed gastrointestinal bleeding. A tracheoinnominate fistula was suspected when there was bleeding around the tracheostomy. The patient underwent a median sternotomy with innominate artery ligation.

The article will discuss the presentation, evaluation, and emergent management of this lethal complication of tracheostomies. The patient survival is dependent on high clinical suspicion, rapid diagnosis, and emergent surgical management.

## Introduction

Tracheoinnominate fistula (TIF) is a rare (0.1%-1%) but a life-threatening complication after tracheostomy. The clinician caring for a patient with a tracheostomy must have a high suspicion for TIF bleedings that occur three days to six weeks after tracheostomy. TIF accounts for 10% of all bleeding associated with tracheostomies. Fifty percent of patients present with a sentinel bleed described as minor bleeding that spontaneously stops. The peak incidence is between seven to fourteen days after the procedure. Risk factors for developing TIFs include chronic steroid use, recent tracheostomy, overinflated cuff, high riding innominate artery, excessive movement of the tracheostomy, or low positioning of the tracheostomy.

## Case presentation

A 63-year-old female with a history of renal transplantation and chronic immunosuppression was admitted to the tertiary care center for respiratory symptoms. During her hospitalization, she progressed to respiratory failure and found to have disseminated histoplasmosis. After failed attempts at extubation, the patient underwent an open tracheostomy. She was discharged to a long-term acute care facility (LTAC).

The patient developed bright red blood per rectum during her stay at the LTAC without evidence of bleeding at the tracheostomy. Her hemoglobin level was 4.5 mg/dL; a blood transfusion was initiated at the facility and she was transferred to the local community hospital's intensive care unit (ICU). During routine morning patient care, the nursing staff noticed minor non-pulsatile bleeding around the tracheostomy and immediately alerted the ICU physician team. On initial inspection, there was no noted bleeding of the exterior surfaces while the patient was positioned with the head of the bed at 30 degrees. Bleeding recurred when the patient was placed back into the supine position, prompting urgent surgical consultation.

The surgical team and the ICU team proceed to evaluate for bleeding sources. A flexible fiberoptic scope was used to investigate the upper airway and no bleeding was seen from her nasopharynx or oropharynx. The tracheostomy was also evaluated and no bleeding was seen distal or proximal to the tracheostomy site, while the tracheostomy cuff was deflated. Due to the recurrence and unidentifiable source of bleeding, surgical evaluation and management were needed. While prepping the patient, pulsatile bleeding was observed from the tracheostomy. The patient started to develop hemodynamic instability and surgical management deemed necessary. Interventional radiology was not considered at this time because the service was not immediately available on site. The cuff was hyperinflated, which stopped the bleeding and the patient was emergently taken to the operating room with the general surgery team. The thoracic surgeon on call was notified and would meet the team in the operating room.

The patient was prepped for surgery. A median sternotomy is used to gain access to the great vessels. The pericardium was opened to provide additional visualization and mobilization of the great vessels. The innominate vein was first mobilized and provided exposure to the innominate artery. The innominate artery's course was traced and fistula palpated on the posterior wall of the artery. Vascular clamps were applied proximal to the fistula and distally, ensuring that the thyrocervical trunk remained intact. The proximal end of the vessel was ligated using a vascular stapler. The distal end was oversewed in two layers using a 4-0 prolene suture. Thymic tissue was mobilized and placed over the tracheal fistula. This method provided quick control and coverage of the defect. The sternotomy was closed and resuscitation continued until the transport team arrived in the operating room to take the patient to the tertiary care center.

## Discussion

TIFs are highly lethal complications associated with a tracheostomy. Early bleeding within minutes to hours after performing a tracheostomy is often from poor hemostasis during the initial tracheostomy or from a coagulopathy. Tracheostomy bleeding occurring three days to six weeks in the postoperative period should be assumed to be a TIF until proven otherwise [1]. Fistulas from surrounding arteries, common carotid, inferior thyroid, thyroid ima, aortic arch, and the innominate vein, have been reported [2]. Risk factors for developing a TIF include steroid use, tracheal infection, tracheostomy below the third tracheal ring, high-riding innominate artery, pressure necrosis from an overinflated tracheal cuff, malposition of the tracheal cuff, poorly sized tracheal appliance, or excess movement of the appliance [2]. Pathology shows the evolution of the fistula progresses from superficial tracheitis to necrosis, loss of cartilage, and subsequent fistulization [3].

Controlling hemorrhage

Quick bedside management of the bleeding should include hyperinflating the tracheal cuff balloon, allowing for temporary control in 85% of the cases and is the first maneuver that should be attempted [1,2,4-7]. Additional methods of bleeding control involve withdrawal of the tracheostomy tube with the advancement of an endotracheal tube past the bleeding to prevent blood collecting in the lungs, and digital compression of the artery against the manubrium by entering the pretracheal space, known as the Utley maneuver [1,7,8]. Bronchoscopy is used to evaluate the presence of the fistula, and imaging studies such as conventional angiography or CT angiography should be used in stable patients. Imaging shows a blush into the trachea, and can be used to diagnose a TIF [7]. Patients should additionally have adequate peripheral access to provide effect resuscitation. The unstable patient may need blood products, and was started on massive transfusion, crystalloid, or vasopressors until the TIF can be definitively managed.

Surgical management

Communication between the anesthesia and surgical team is crucial while preparing the patient for surgical intervention. The anesthesia team evaluates for bleeding in the airway while the tracheostomy cuff is deflated and the appliance is withdrawn. An endotracheal tube is advanced under visual guidance past the suspected source of fistulization. This placement helps prevent blood from entering the distal airway. Additional hemodynamic monitoring devices and vascular access lines are commonly placed.

A median sternotomy is used to gain access to the great vessels. The innominate artery is the first branch of the aorta and gives rise to the thyrocervical trunk. The innominate vein is mobilized, and the pericardium may need to be opened to obtain sufficient exposure. The innominate artery is then followed distally until it crosses over the trachea. Careful blunt dissection will reveal the fistula posterior to the innominate artery.

**Figure 1:**
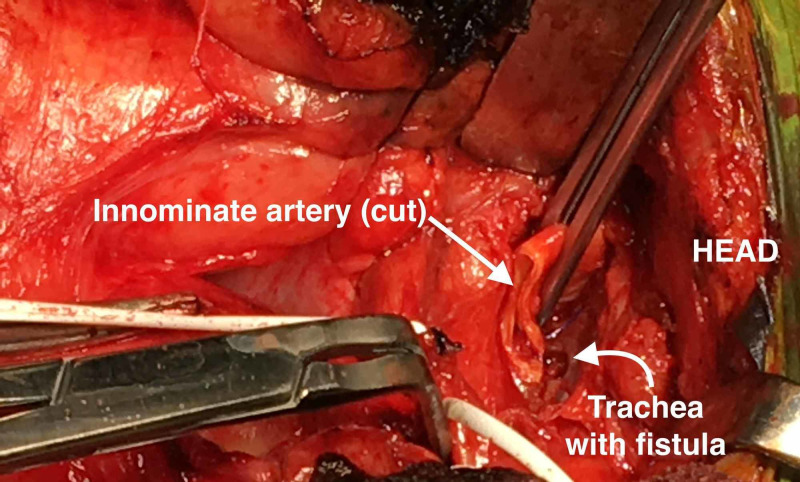
Clamped innominate artery with tracheal fistula exposed

Once the TIF is identified, vascular clamps are placed to obtain control of the innominate artery (Figure 1). The proximal end of the innominate artery is ligated with a vascular stapler. The distal end of the innominate artery or the thyrocervical stump can be stapled or suture ligated in an oversewn fashion. When the artery is ligated, there is a sharp sudden drop in the right arm arterial pressure.

The trachea may be primarily repaired with the PDS suture after debridement of devitalized tissue or a bovine pericardial patch may be necessary. A lesser documented, but viable thymic flap can be used to buttress the tracheal opening and the innominate artery stumps to prevent fistulization. Other options include using omentum and the sternocleidomastoid muscle.

The endotracheal tube is left in place, the sternotomy is closed, and the patient is maintained in the ICU for continued neurovascular monitoring.

Intra-operative and postoperative considerations

Arterial lines placed in the right arm will demonstrate decreased perfusion when the innominate artery is ligated. It is vital that the placement of a hemodynamic monitoring device should take this effect into consideration. Checking the stump pressure of the distally ligated innominate artery will provide an early indication of retrograde flow to the right upper extremity and cerebral hemisphere.

Postoperatively, the patient should be monitored for ongoing neurovascular changes with special attention to the right upper extremity and right brain hemisphere perfusion. Perfusion to these areas is dependent on retrograde flow from the left brain hemisphere through a patent Circle of Willis.

Long-term outcomes show an overall poor prognosis for those patients who develop a TIF and often the cause of death for survivors is due to their other comorbidities. Innominate artery ligation has shown up to a 10% incidence of neurological deficit. Patients should be monitored for these changes. Perfusion to the brain and right upper extremity relies on the retrograde flow through the Circle of Willis.

## Conclusions

TIFs are highly lethal if not addressed in a timely manner, and physicians caring for a patient with a tracheostomy should have a high suspicion for a TIF after the immediate three to five days postoperative period. Successful management includes immediate bedside maneuvers such as hyperinflating the tracheostomy balloon, or the Utley maneuver as well as utilizing interventional radiology, endovascular treatment for a stable patient, or surgical management for an unstable patient or when minimally invasive procedures are not immediately available. Communication between the surgeon, anesthesia, and critical care team is vital to isolate the fistula, providing ongoing hemodynamic and neurological monitoring.
